# Mechanism of CXCL8 regulation of methionine metabolism to promote angiogenesis in gliomas

**DOI:** 10.1007/s12672-024-01467-2

**Published:** 2024-11-02

**Authors:** Jie Chang, Yi Pan, Fengfeng Jiang, Wenxia Xu, Yue Wang, Lude Wang, Bin Hu

**Affiliations:** 1 Central Laboratory, Affiliated Jinhua Hospital, Zhejiang University School of Medicine, Jinhua, Zhejiang China; 2 Precision Diagnosis and Treatment Center, Affiliated Jinhua Hospital, Zhejiang University School of Medicine, Jinhua, Zhejiang China; 3 Key Laboratory of Nutrition and Metabolism Research for Oncology, Affiliated Jinhua Hospital, Zhejiang University School of Medicine, Jinhua, Zhejiang China; 4 Neurological Surgery Department, Affiliated Jinhua Hospital, Zhejiang University School of Medicine, Jinhua, Zhejiang China; 5 Dian Diagnostics Group Co. Ltd, Hangzhou, China; 6 Department of Pathology, Affiliated Jinhua Hospital, Zhejiang University School of Medicine, Jinhua, Zhejiang China

**Keywords:** Glioma, Methionine metabolism, CXCL8, Angiogenesis

## Abstract

**Background:**

Gliomas are the most common malignant brain tumors characterized by angiogenesis and invasive growth. A detailed understanding of its molecular characteristics could provide potential therapeutic targets. In the present study, we sought to **e**xplore the key gene CXCL8 in methionine metabolism in gliomas and its potential role in angiogenesis.

**Methods:**

U251 glioma cells were divided into control and methionine-restriction tolerant (constructed with 1/4 of the standard level of methionine in the culture medium) groups for transcriptome and metabolome analysis. To confirm the functions and mechanism of CXCL8 in glioma, heat map, volcano map, Go enrichment, gene set enrichment analysis (GSEA), protein–protein interaction network analysis, RT-PCR, western blotting assays, chicken embryo chorioallantoic membrane (CAM) test, chicken embryo yolk sac membrane (YSM) test and transplantation tumor nude mice model were performed. The TCGA database, CGGA database and clinical tissue samples were used to analyze CXCL8’s significance on prognosis for patients with glioma.

**Results:**

CXCL8 expression was significantly up-regulated in methionine-restricted tolerance cells, it also activated vascular system development and triggered angiogenesis. CXCL8 expression is negatively correlated with survival prognosis in gliomas.

**Conclusions:**

Glioma cells promote angiogenesis in methionine-restricted environments through the activation of CXCL8, compensating for nutrient deprivation, and possibly contributing to the failure of antiangiogenic therapy.

**Supplementary Information:**

The online version contains supplementary material available at 10.1007/s12672-024-01467-2.

## Introduction

Gliomas are one of the most common primary brain tumors with limited surgical and medical chemotherapy, resulting in high recurrence and mortality rates [1, 2]. Due to the extremely abundant blood vessels in glioma tissues, medical researchers have developed treatments that target and inhibit angiogenesis. Antiangiogenic drugs, such as bevacizumab, have not significantly prolonged the overall survival of patients with glioma [3]. A dilemma facing experts and scientists in glioma research is the high dependence of tumor growth on angiogenesis and the failure of antiangiogenic treatment [4, 5]. Consequently, understanding the molecular mechanisms behind the resistance to antiangiogenic therapy in gliomas will help develop new therapeutic strategies.

The rapid proliferation of tumor cells requires a large amount of nutrients, whereas a poor blood supply makes the microenvironment nutrient-deficient. As a part of metabolic remodelling, tumor cells can meet energy and material requirements, such as aerobic glycolysis [6–8]. Glioma cells are highly dependent on methionine, among many nutrients [9]. Typically, brain cells grow slowly and have a lower requirement for methionine. In contrast, glioma cells require a higher demand for methionine during rapid proliferation, a phenomenon known as the Hoffman effect [10]. Clinically, PET-CT with ^11^C-labelled methionine (^11^C-Met PET) is performed to diagnose and stage gliomas based on this principle [11]. Dietary restriction of methionine intake, which is an essential amino acid, is a potential anticancer strategy [12, 13]. How do glioma cells adapt to the methionine-deprived microenvironment to proliferate? Through investigating this question, new strategies will be developed to improve the efficacy of antiangiogenic therapy in gliomas.

CXC chemokine ligand 8 (CXCL8), an important member of the chemokine family, plays a key role in immune microregulation, angiogenesis, and tumorigenesis [14–16]. We previously found that CXCL8 was significantly upregulated in glioma cells with a methionine-restricted microenvironment [17]. Nevertheless, it remains unclear how CXCL8 affects methionine metabolism and angiogenesis and its relationship to the prognosis of glioma patients. Therefore, the present study aims to investigate the mechanism of action of glioma cells in a methionine-restricted environment by activating the transcriptional expression of CXCL8 to promote angiogenesis. Analyzing transcriptomic sequencing data from methionine-restricted tolerant cells and combining in vitro and in vivo experiments will provide a new perspective on metabolic remodelling processes during antivascular therapy and explore possible intervention targets.

## Materials and methods

1. Cell culture and grouping methods: Human-derived glioma cell lines U251MG and U87MG and umbilical vein endothelial cell line HUVEC were purchased from Shanghai Academy of Sciences cell bank. Cells were inoculated in 1640 medium and randomly divided into control group, methionine restriction-tolerant group (M group), and methionine restriction-tolerant + SB225002 group (M + SB225002 group), in which the M group was constructed as follows: RPMI-1640w/o amino acid medium powder containing 19 amino acids were added as a standard, and the amount of methionine was reduced from 15 to 3.75 mg/L[17, 18] to prepare methionine-restricted medium (MSM), and cells cultured in MSM for ~ 20 days were termed as U251-M or U87-M; the M + SB225002 group was the U251-M or U87-M cells combined with the CXCL8–CXCR2 signaling axis inhibitor SB225002 treatment group. All cells were cultured at 37 °C in a 5% CO_2_ incubator and passaged by 2.5 g/L trypsin digestion.
2. Reagents and antibodies: 1640 medium and fetal bovine serum were purchased from Gibco, trypsin was purchased from Zhejiang GinoSebail Biotechnology Co., Ltd, matrix gel was purchased from Shanghai Yisheng, CXCL8–CXCR2 signal axis inhibitor SB225002 (HY-16711) was purchased from MCE, bevacizumab was purchased from Roche, Germany, and antibodies against CXCL8 (94407), VEGFA (50661), and β-actin (4967) were purchased from Cell Signaling Technology, USA.
3. Glioma datasets: In this study, two transcriptome sequencing datasets from previous studies were acquired: one is the U251 and methionine-restricted tolerant cells U251-M dataset for human-derived glioma cells [18], and the other is the U251-M and SB225002-treated U251-M cells dataset[17]. In addition, we obtained three TCGA glioma cohorts, TCGA-GBM, TCGA-LGG and the integrated TCGA-GBMLGG from the UCSC database (https://xenabrowser.net/) [19] as well as from the China Glioma Genome Atlas database (http://www.cgga.org.cn/download.jsp) three glioma cohorts CGGA-301, CGGA-325 and CGGA-693 were obtained for subsequent analysis [20].
4. Differentially expressed gene analysis: The R package “limma” [21] was used to identify differentially expressed genes (|logFC|> 2 and FDR < 0.05) between the experimental and control groups. Functional enrichment analysis of the differentially expressed genes was performed by Gene Ontology (GO) [22] and Kyoto Encyclopedia of Genes and Genomes (KEGG) [23] to assess the biological significance of the differentially expressed genes. GSEA was then performed using the R package “clusterProfiler” [24, 25], in which the background gene sets were downloaded from the GSEA website (https://www.gsea-msigdb.org/).
5. Construction of protein–protein interaction network and screening of core genes: Protein–protein interaction network comprises proteins that participate in various aspects of life processes such as biological signaling, gene expression regulation, energy and material metabolism, and cell cycle regulation through their interactions with each other. In this study, protein interaction network analysis (confidence level > 0.4) was performed through the online website STRING (https://cn.string-db.org/), followed by screening of hub (hub) genes using the cytohubba plug-in of Cytoscape 3.7.2 [25] software.
6. Western blotting assays: U251 cells were cultured under MSM conditions for 0–5 days, and then total cellular proteins were extracted, BCA protein quantification kit was used to determine the protein concentration, and then SDS-PSGE electrophoresis was carried out (80 V, 2 h), and the gels were transferred to PVDF membranes at 250 mA for 2 h, and 50 g/L skimmed milk powder was added. After sealing, anti-CXCL8 or VEGFA rabbit polyclonal antibody (1:1000) was added and incubated overnight. HRP-labelled goat anti-rabbit IgG was incubated for 1 h, and then ECL enhancement solution was added to obtain images by exposing the gel in a BIO-RAID gel imaging system, with β-actin as the internal reference.
7. RT-PCR: Total RNA was extracted from cell samples using Trizol reagent (Life Technologies, CA, United States), and 1 μg of total RNA was reverse transcribed to cDNA using RT kit (TaKaRa). 1 μg of total RNA was amplified on a Light Cycler 480 II PCR instrument. The relevant genes were amplified on a Light Cycler 480 II PCR instrument. The level of CXCL8 mRNA was calculated using the standard 2^−ΔΔCt^ relative quantification method (using β-actin as an internal reference).

The primer sequences are as follows:

CXCL8 Forward 5′-AGCTCTGTGTGAAGGTGCAG-3′

             Reverse 5′- TTCCTTGGGGTCCAGACAGA-3′

β-actin Forward 5′-ACTCTTCCAGCCTTCCTTCC-3′

            Reverse 5′- CGTCATACTCCTGCTTGCTG-3′

8. Chorioallantoic membrane (CAM) test: Chicken embryos were incubated for 7 days, after which they were incubated at 37℃ and 55% humidity for 24 h. Next, open a 1 cm × 1 cm hole in the top of the air chamber, remove the shell membrane with sterile forceps, place a silica gel ring (10.1 mm inner diameter; 12 mm outer diameter) and add U251 cell suspension (2 × 10^6^ cells/100 μL culture medium) dropwise. Immediately closed chicken embryos with sterile tape and put into the incubator to continue incubation for 7–10 days. The control group was treated with 0.9% NaCl (30 μL/chicken embryo), and the experimental group was treated with 10 μM SB225002 (30 μL/chicken embryo). After 1–3 days of culture, CAM vessels were photographed, stripped, and recorded.
9. Yolk sac membrane (YSM) test: Chicken embryos were incubated for 7 days at 37℃ and 55% humidity for 24 h and then open a 1 cm × 1 cm hole in the top of the air chamber, after removing the shell membrane with sterile forceps, placed the silica gel ring (10.1 mm inner diameter; 12 mm outer diameter) in the yolk sac vascular part of the chicken embryo. 50 μL of 0.9% NaCl, SB225002 (10 μM) or bevacizumab (10 μM) was placed into the chick embryos with well-developed blood vessels. After 24 h of incubation, Pictures were taken, and the results were recorded.
10. Tubulogenesis test: Add 50μL of matrix gel into the pre-cooled 96-well plate, put it into the incubator for 30 min to make it solidify, plant HUVEC or U87 cells (5000/100μL/well) on the matrix gel and culture them for 2 h, added conditioned medium with methionine to culture cells to 0, 1, 3, 5 days, and cultured them at 37℃ for 6-8 h, and then under the optical microscope Observe and take photos to record the tube formation (3 compound wells were set in each group).
11. Tumor mice models: All studies involving animals were performed according to and approved by the Animal Care and Use Committee of Jinhua Food and Drug Inspection and Testing Institute (approval number: AL-JSYJ202232). Nude mice (female 4 weeks old) were used for tumor-bearing model studies. 1 × 10^6^ U251 cells in 100 μL 1640 medium without FBS or antibodies were injected subcutaneously and placed on indicated diets. Tumor-bearing mice were placed randomly on control and methionine-restricted diets (1/10 standard methionine diets) for about 3 weeks, and the mice were sacrificed, and tumor tissue subsequently resected after 3 weeks of inoculation.
12. Immunohistochemical (IHC) staining: The obtained tumor tissues were fixed in 10% formaldehyde, embedded in conventional paraffin and sectioned. The slices were baked at 60 ℃, deparaffinized in conventional xylene, rehydrated with gradient ethanol, repaired with high pressure in citrate buffer to restore the antigen, and then sealed in 3% hydrogen peroxide, then stained and blocked by dropwise addition of CD31 antibody (diluted in 1:200), VEGFA antibody (diluted in 1:200), and HRP-labelled goat-anti-rabbit IgG secondary antibody. The samples were stained and blocked in 3% hydrogen peroxide. Positive staining was light yellow, yellow and brownish yellow.
13. Metabolomics assay: UPLC-ESI–MS/MS was used for qualitative and quantitative detection of the target metabolites, and the specific conditions and analytical methods were as follows: chromatographic method: injection volume: 1 μL; flow rate: 0.3 mL/min; mobile phase: A (0.2% formic acid–water solution containing 10 mM ammonium formate), B (0.2% formic acid-90% acetonitrile water containing 10 mM ammonium formate); gradient elution method: 0 min A/B (0:100, V/V), 2 min A/B (0:100, V/V), 3 min A/B (10:90, V/V), 15 min A/B (15:85, V/V), 18 min A/B (60:40, V/V), 19 min A/B (60:40, V/V). 19.01 min A/B (0:100, V/V), 20 min A/B (0:100, V/V). Mass spectrometry method: gas curtain gas: 35 (psi); collision-induced ionisation parameter: medium; positive ion spray voltage: 5500 V; negative ion spray voltage: − 4500 V; ion source temperature: 450 °C; column temperature: 40 °C; spray gas (Gas1): 55 (psi); auxiliary heating gas (Gas2): 55 (psi).
14. Statistical analysis: Kaplan–Meier curves were plotted in this study using the R package “survival” [26] to analyse the survival of patients with high and low CXCL8 expression in the glioma cohort. The R package “forestplot “ was used to analyse the relationship between CXCL8 and prognosis in the TCGA pan-cancer cohort. Glioma datasets analysis was performed in R (version 3.6.1), and *P* < 0.05 was considered statistically significant. For RT-PCR assays, an unpaired t test was analyzed. For IHC quantification analysis, we counted the number of positive cells in 5 random fields of view at 20× and averaged them to get the number of positive cells in this film and analyzed positive ratios statistically. For tube formation studies, one-way ANOVA with multiple comparisons was used to determine *P* value. For CAM studies, an unpaired t test was performed to determine the statistical difference in tumor weight. The results for western blotting assays were representative of at least three biological independent experiments. All statistical analyses and visualization were performed by GraphPad (Prism 9) or R (version 3.6.1). Data were reported as the mean ± SD and we use ns (*P* > 0.05), *(0.01 < *P* < 0.05), **(0.001 < *P* < 0.01) and ***(*P* < 0.001) to indicate the levels of *P* value.

## Result

### Transcriptomic analysis of methionine-restricted tolerant cells revealed a significant enrichment of angiogenic pathways

**Fig. 1:**
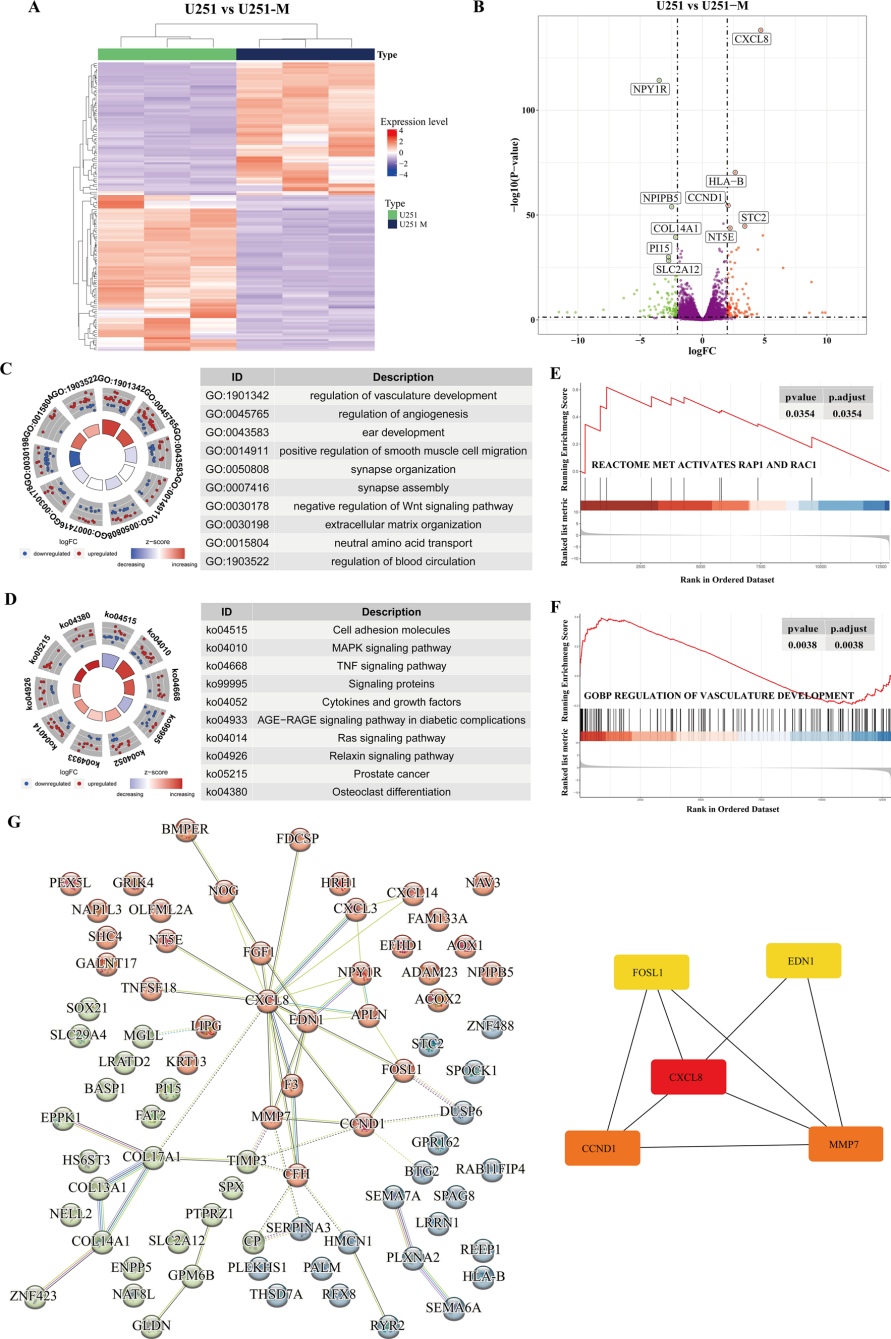
Transcriptomic analysis of methionine restriction tolerant cells. **A** Heat map of differentially expressed genes in U251 and U251-M cells. **B** Volcano map of differentially expressed genes in U251 and U251-M cells. **C** Results of GO enrichment analysis of differentially expressed genes in U251 and U251-M cells. **D** KEGG enrichment of differentially expressed genes in U251 and U251-M cells analysis results. **E** GSEA enrichment analysis showed that methionine deprivation activates rap1 and rac1 signaling pathways. **F** GSEA enrichment analysis showed that methionine deprivation regulates the development of vascular system. **G** Protein–protein interaction network analysis. The left panel shows the protein–protein interaction network of differential genes, and the right panel shows the top five ranked hub genes

In the previous study, we configured the medium with a methionine content of 1/4 of the standard content and constructed a subpopulation of stably growing cells, U251-M [18]. To gain insight into the differential gene expression profiles of U251-M and U251 cells, we performed transcriptome sequencing analysis, which yielded a total of 186 differential genes, of which 87 were up-regulated, and 99 were down-regulated (Fig. 1A, Table S1). The five most significantly up-regulated genes were CXCL8, human leukocyte antigen B (HLA-B), cell cycle protein D1 (CCND1), skatolein 2 (STC2), and extracellular 5'-nucleotidase (NT5E), and the five most significantly down-regulated genes were neuropeptide Y1 receptor (NPY1R), nuclear pore complex interactions protein B5 (NPIPB5), collagen XIVα1 chain (COL14A1), peptidase inhibitor 15 (PI15), and glucose transporter protein 12 (SLC2A12) (Fig. 1B). In order to explore the underlying biological mechanisms occurring after methionine deprivation, we performed GO functional enrichment analyses in terms of biological processes (BP), molecular functions (MF), and cellular composition (CC). Functional annotation was performed in three aspects: BP results were mainly enriched in the regulation of vascular development, regulation of angiogenesis, regulation of haematopoiesis, positive regulation of smooth muscle cell migration, negative regulation of the Wnt signaling pathway and regulation of haematopoiesis; MF results were enriched in neutral amino acid transporter activity, MAP kinase phosphatase activity and heparin binding; and CC results were enriched in the lumen of the endoplasmic reticulum, synaptic membranes and the extracellular matrix (Fig. 1C, Table S2). KEGG pathway enrichment analysis showed that differential genes were enriched in the MAPK signaling pathway, TNF signaling pathway and Ras signaling pathway, among others (Fig. 1D, Table S3). In addition, further gene set enrichment analysis (GSEA) showed that methionine restriction activates the rap1 and rac1 signaling pathways and is associated with the developmental regulation of the vascular system (Fig. 1E, F). For the differential genes, we performed PPI analysis (threshold > 0.4) via the online website STRING. We imported the results into Cytoscape software to analyse the hub genes during methionine restriction tolerance using the cytoHubba module. The results showed that the top five hub genes most affected under the methionine-restricted environment were CXCL8, CCND1, matrix metalloproteinase 7 (MMP7), endothelin 1 (EDN1), and FOS-like antigen 1 (FOSL1) (Fig. 1G). These results suggest that, in the face of a methionine-deprived microenvironment, glioma cells promote angiogenesis through the possible secretion of CXCL8 to compensate for nutrient supply.

### CXCL8 expression is negatively correlated with survival prognosis in gliomas

**Fig. 2:**
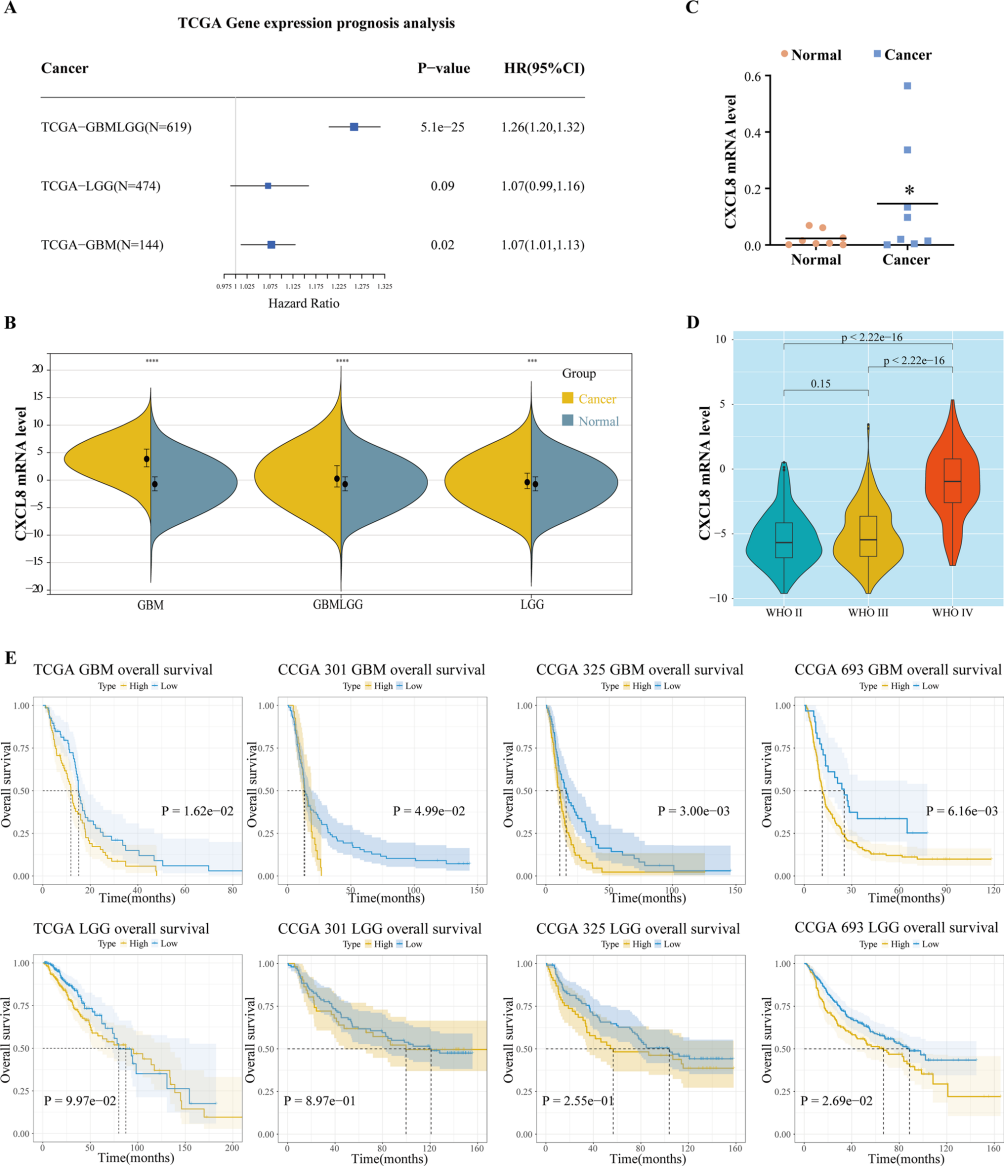
Clinical information analysis of CXCL8 in gliomas. **A** Forest plot of the relationship between CXCL8 and prognosis in the TCGA glioma cohort. **B** CXCL8 expression plots of normal and tumor samples in the TCGA glioma cohort. **C** RT-PCR assay for detecting CXCL8 mRNA expression levels in glioma tissues and paracancer tissues. **D** Violin plots depicting the distribution of CXCL8 expression levels in TCGA glioma cohorts, stratified by different WHO staging categories for CXCL8 expression level distribution. **E** Kaplan–Meier curves of CXCL8 gene expression versus overall patient survival in glioma cohorts of TCGA and CGGA

To further investigate the prognostic role of CXCL8 in gliomas, we downloaded the uniformly standardized brain glioma dataset from the UCSC database and analyzed the relationship between CXCL8 expression and survival prognosis. The results showed that high CXCL8 expression was a risk factor for morbidity in cohorts TCGA-GBMLGG (HR = 1.26, *P* = 5.1e−25) and TCGA-GBM (HR = 1.07, *P* = 0.02) (Fig. 2A). Meanwhile, we found that in the TCGA glioma cohort, CXCL8 expression was significantly higher in tumor samples than in normal samples (Fig. 2B). Next, we collected pathology specimens from eight patients with glioma and used RT-PCR to detect and compare the expression of CXCL8 in glioma tissues and paracancerous tissue specimens and found that the positive expression rate of CXCL8 mRNA was significantly higher in glioma tissues compared with paracancerous tissues (Fig. 2C), which indicated that the high expression of CXCL8 might be associated with poor prognosis. To investigate whether CXCL8 differed in GBM and LGG, we examined the CXCL8 expression levels in patients with gliomas of different WHO stages and found that CXCL8 expression was not significantly different between WHO II and WHO III (*P* = 0.15) but was significantly higher between WHO II and WHO IV (*P* < 2.22e−16) and between WHO III and WHO IV (*P* < 2.22e−16) were significantly different (Fig. 2D), suggesting that there was a significant expression difference of CXCL8 between low-grade gliomas and high-grade gliomas. To further explore the relationship between CXCL8 and glioma survival, we divided the glioma cohort from the TCGA database and the three glioma cohorts from the CGGA database into high-expression and low-expression groups based on the expression value of CXCL8 and compared the prognosis between them, respectively. The results of the survival curves showed that in high-grade gliomas, the survival of the high-expression group of CXCL8 was significantly shorter (Fig. 2E), whereas in low-grade gliomas, the high-expression CXCL8 group showed a poorer prognosis only in the CGGA-693 cohort. These database analyses suggest that CXCL8 expression levels are negatively correlated with survival prognosis in high-grade gliomas and may predict poor prognosis in high-grade gliomas.

### Effect of methionine-restricted environment on CXCL8 expression and angiogenesis

**Fig. 3:**
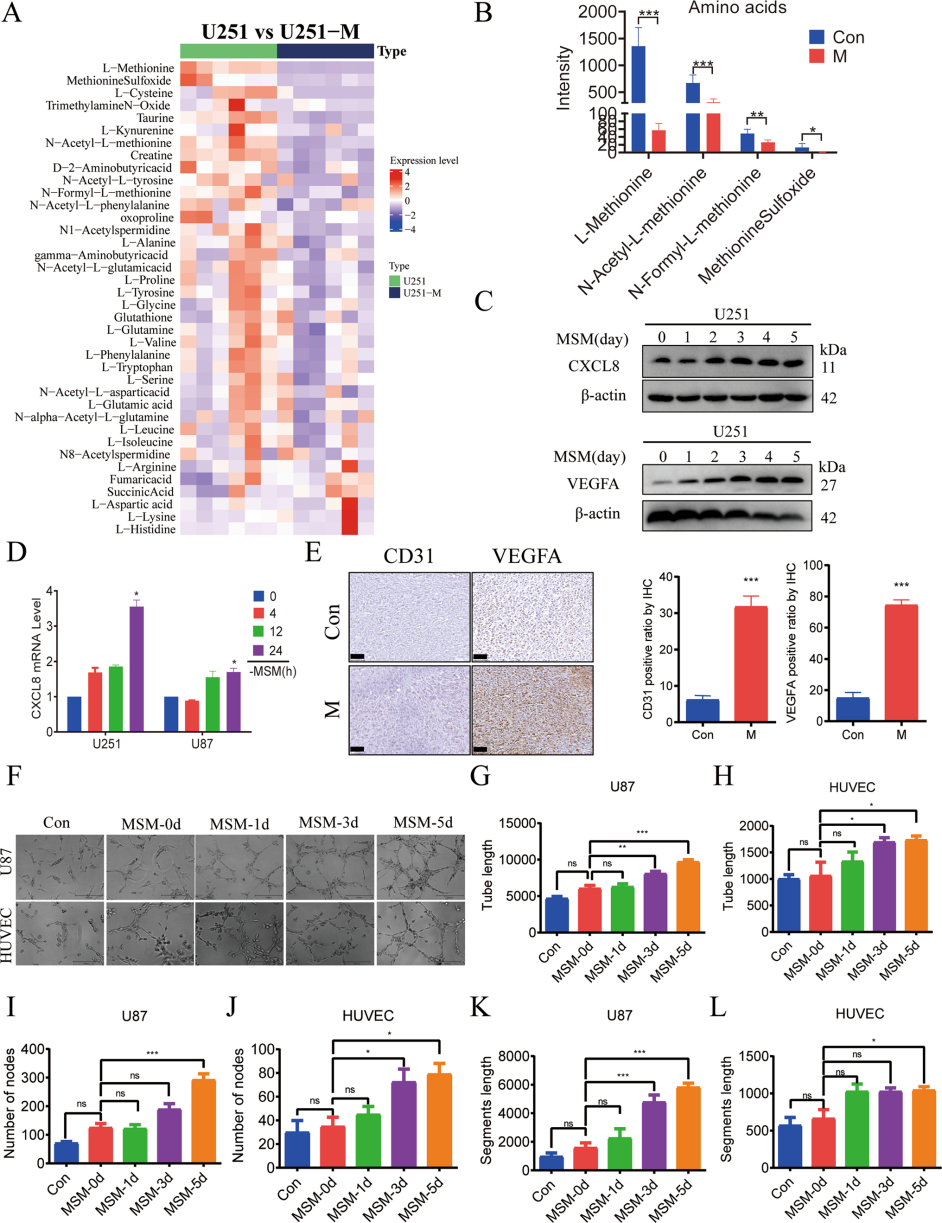
Effect of methionine-restricted environment on CXCL8 expression and angiogenesis. **A** Heat map of metabolomics analysis of targeted amino acids in control and methionine-restricted tolerant cell groups. **B** Statistical plots of the levels of l-methionine and its derivatives, *N*-acetyl-l-methionine, *N*-formyl-l-methionine, and methionine sulfoxide, for control and methionine-restricted tolerant cell groups (*P < 0.05,**P < 0.01,***P < 0.001). **C** Western blotting to detect the expression of CXCL8 and VEGFA proteins in U251 cells. **D** RT-PCR to detect the expression level of CXCL8 mRNA in methionine-restricted cultured cells after 0, 4, 12, and 24 h (*P < 0.05). **E** Control and methionine-restricted diet group nude mice transplanted tumor tissues immunohistochemical staining of CD31, VEGFA, scale bar = 50 μm. F. Tubulogenesis assay to detect the effect of methionine-restricted culture on the tubulogenic ability of HUVEC, U87. **G**–**L** Graphs of statistical analysis of tube length, number of nodes, and segments length as compared with MSM-0d (*P < 0.05,**P < 0.01)

Targeted amino acid metabolomics results (Fig. 3A, B) showed that the amino acid metabolic profile of methionine-restricted tolerant U251 cells was significantly altered, in which the levels of l-methionine and its derivatives, *N*-acetyl-l-methionine, *N*-formyl-l-methionine, and methionine sulfoxide were reduced compared with the control group. The WB results showed that upon deprivation of methionine in the culture medium, with the time of prolongation, the expression of CXCL8 and pro-angiogenic factor VEGFA increased significantly (Fig. 3C). RT-PCR results showed (Fig. 3D) that the relative expression of CXCL8 mRNA increased with the prolongation of methionine-restricted culture. Immunohistochemistry was applied to detect the expression of CD31 and VEGFA proteins in the tumor tissues, and the results showed that the expression levels of CD31 and VEGFA in the tumor tissues of the methionine-restricted diet group were significantly higher than those of the control group (Fig. 3E). In vitro tube formation experiments using umbilical vein endothelial cells HUVEC as well as glioma cells U87, which have a strong tube-forming ability, revealed that methionine-restricted culture conditions enhanced tube formation, as evidenced by an increase in the length of the formed tubules, the number of nodes, and the length of the backbone (Fig. 3F–L).

### Effect of inhibition of CXCL8 expression on angiogenesis in glioma

**Fig. 4:**
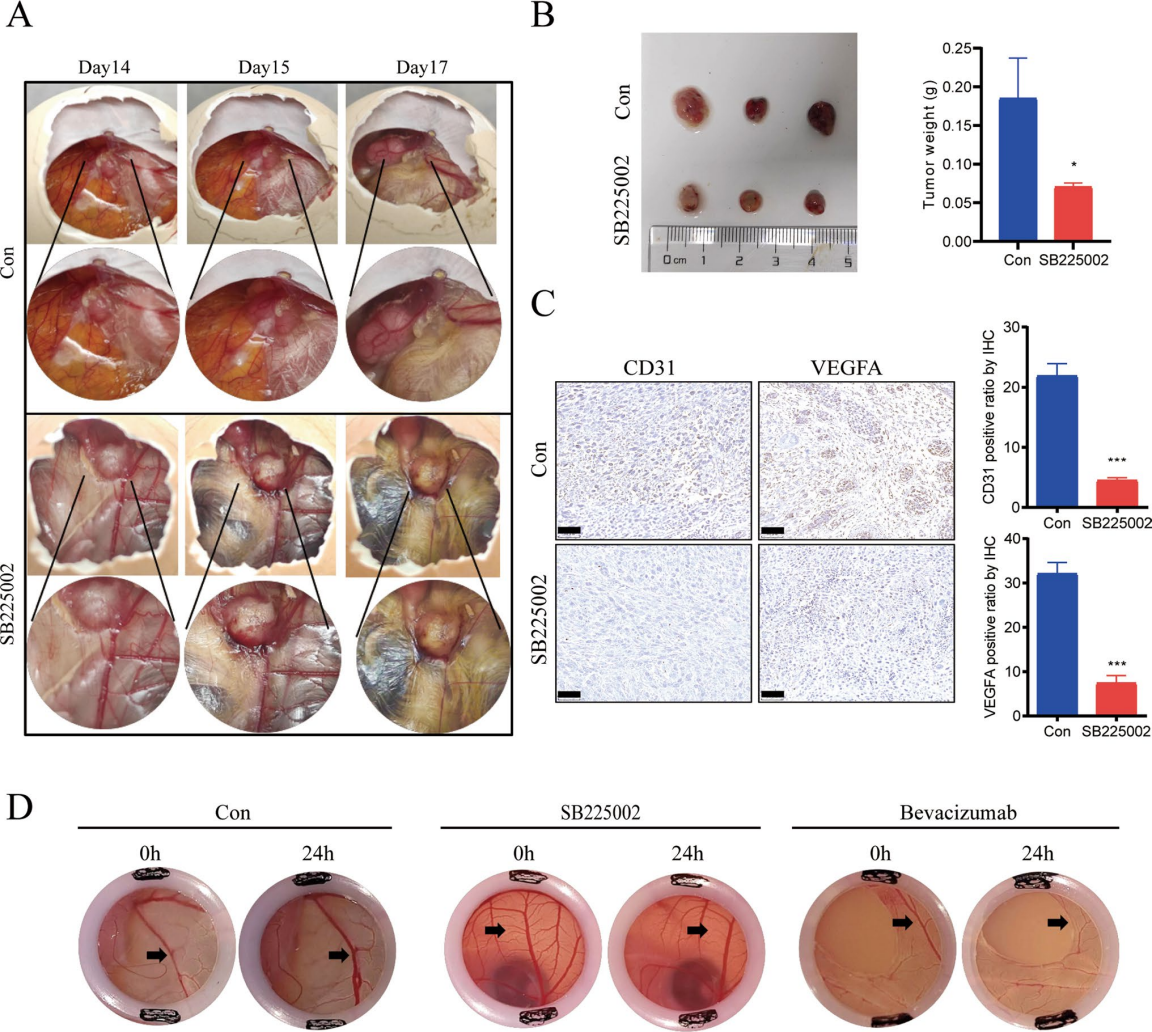
Effect of inhibiting CXCL8 expression on glioma angiogenesis. **A**, **B** CAM experiments to determine the effect of CXCL8–CXCR2 signaling axis inhibitor SB225002 treatment on graft tumor angiogenesis. **C** Immunohistochemical staining of nude mice graft tumor tissues for CD31, VEGFA, scale bar = 50 μm. **D** YSM experiments to determine the effect of SB225002 and positive control bevacizumab treatment on angiogenesis

Angiogenesis is indispensable for maintaining the growth of gliomas, and we further explored the role of high CXCL8 expression during methionine metabolism on angiogenesis. A graft tumor model was constructed in chick embryo chorionic allantoic membrane (CAM), and treatment with the CXCL8–CXCR2 signaling axis blocker SB225002 showed a significant decrease in graft tumor vascular density and in tumor weight (Fig. 4A, B). In the methionine-restricted microenvironment, when CAM was added to the CXCL8–CXCR2 signaling axis blocker SB225002, the immunohistochemical results showed reduced levels of CD31 and VEGFA expression and diminished pro-angiogenic capacity (Fig. 4C). Compared with bevacizumab as an antiangiogenic positive control, treatment with CXCL8–CXCR2 signaling axis blocker SB225002 in a methionine-restricted microenvironment resulted in significant inhibition of vascular plexus growth (Fig. 4D).

### Transcriptomic analysis of the inhibition of the CXCL8–CXCR2 signaling axis

**Fig. 5:**
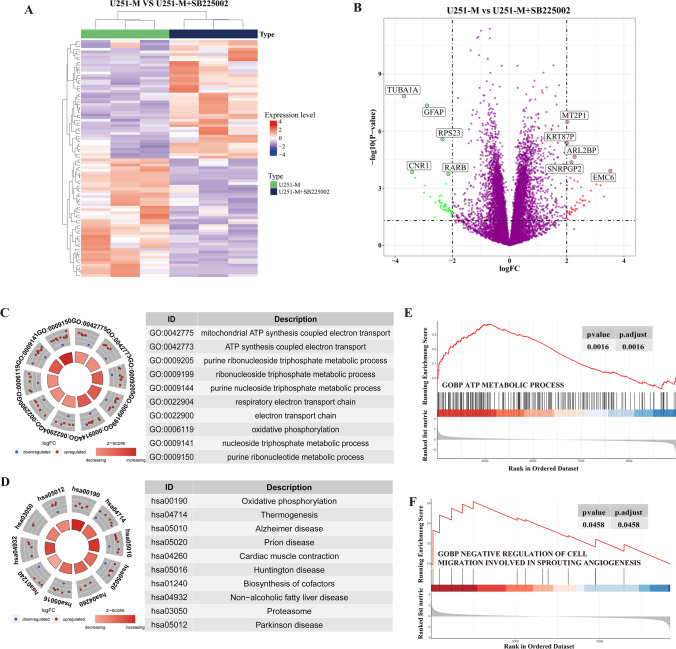
Transcriptomic analysis of the inhibition of the CXCL8–CXCR2 signaling axis in methionine-restricted tolerant cells. **A** Heat map of differentially expressed genes in U251-M and U251-M cells treated with SB225002. **B** Volcano map of differentially expressed genes in U251-M cells and U251-M cells treated with SB225002. **C** GO enrichment analysis of differentially expressed genes in U251-M cells and GO enrichment analysis results of differentially expressed genes in U251-M cells treated with SB225002. **D** KEGG enrichment analysis results of differentially expressed genes in U251-M and U251-M cells treated with SB225002. **E** GSEA enrichment analysis showed that inhibiting of the CXCL8–CXCR2 signaling axis in U251-M glioma cells would activate ATP metabolic processes. **F** GSEA enrichment analysis showed that inhibition of the CXCL8–CXCR2 signaling axis in U251-M glioma cells activates negatively regulated cell migration and angiogenesis

Based on the critical role of CXCL8 in the metabolic remodelling of methionine in gliomas, we further used transcriptome sequencing to explore the gene expression changes involved in blocking the CXCL8–CXCR2 signaling axis in methionine-restricted tolerant cells[17]. By differentially expressed gene analysis, we obtained 413 differentially expressed genes, of which 234 were up-regulated and 179 were down-regulated (Fig. 5A). The five most significantly up-regulated genes were pseudo-metallothionein 2 gene 1 (MT2P1), pseudo-keratin 87 (KRT87P), ADP ribosylation factor-like binding protein 2 (ARL2BP), small nuclear ribonucleoprotein polypeptide G pseudogene 2 (SNRPGP2) and ER membrane protein complex subunit 6 (EMC6). The five most significantly down-regulated genes were microtubulin α1a (TUBA1A), glial fibrillary acidic protein (GFAP), ribosomal protein S23 (RPS23), cannabinoid receptor 1 (CNR1), and retinoic acid receptor β (RARB) (Fig. 5B). GO functional enrichment analysis revealed that metabolism-related signaling pathways, such as ATP-synthesis-coupled electron transport, electron transport chain, oxidative phosphorylation, and nucleoside triphosphate metabolism were enriched (Fig. 5C). KEGG enrichment analysis showed that differential genes were enriched for pathways such as oxidative phosphorylation and thermogenesis (Fig. 5D). GSEA results showed that inhibition of CXCL8 in a methionine-restricted environment activated ATP metabolic processes and inhibited angiogenesis (Fig. 5E, F). These results suggest that blockade of the CXCL8–CXCR2 signaling axis disrupts the signaling network of glioma cells adapted to the methionine-restricted microenvironment and may inhibit angiogenesis by altering energy metabolic pathways such as oxidative phosphorylation.

### Targeted metabolomics analysis of the CXCL8–CXCR2 signaling axis inhibition

**Fig. 6:**
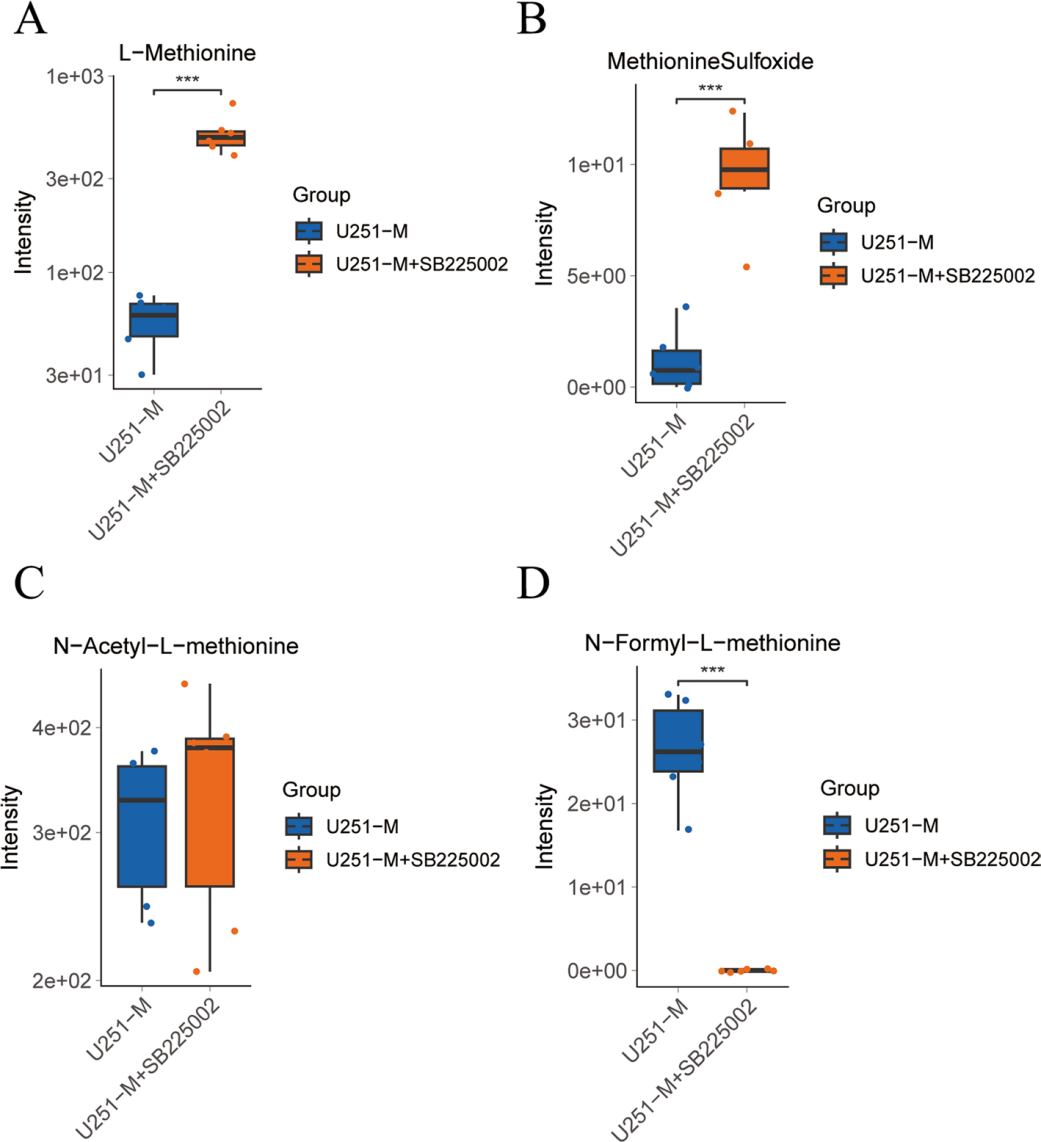
Targeted metabolomic analysis of the CXCL8-CXCR2 signaling axis inhibition in methionine-restricted tolerant cells. **A**–**D** Targeted amino acid metabolomic approach to detect the levels of l-methionine (**A**), methionine sulfoxide (**B**), *N*-acetyl-l-methionine (**C**), *N*-formyl-l-methionine (**D**) in U251-M cells and U251-M + SB225002 cells

Based on the critical role of CXCL8 in the metabolic remodelling of methionine in gliomas, we further used metabolomics targeting 100 amino acids to explore the changes in the metabolism of amino acids involved in the CXCL8–CXCR2 signaling axis. By differential metabolite analysis, the changes in metabolic levels of methionine and its derivatives were focused, in which the levels of L-methionine (Fig. 6A) and its derivative methionine sulfoxide (Fig. 6B) were elevated after inhibition of the CXCL8–CXCR2 signaling axis compared to those in U251-M cells (***P < 0.001). Whereas the level of N-acetyl-L-methionine (Fig. 6C) was not significantly altered, the level of N-formyl-L-methionine (Fig. 6D) was decreased (***P < 0.001) compared with that in U251-M cells.

## Discussion

Gliomas are the most common primary malignant intracranial tumors characterized by massive angiogenesis [27–29]. Despite being widely used in tumor therapy, antiangiogenic strategies have had limited success in the treatment of gliomas. Antiangiogenic drugs like bevacizumab reduce oxygen and nutrient supply to the tumor microenvironment [30]. Tumor cells have been discovered to facilitate cell proliferation and tumor progression through metabolic reprogramming [31, 32]. Therefore, molecular mechanisms of resistance to antivascular therapy areessential for the development of new strategies for gliomas.

Recent studies have found that methionine restriction inhibits tumor cell growth and metastasis [12] and prolongs tumor mouse survival [33]. It remains unclear how methionine metabolism contributes to glioma development and how it interacts with clinical treatment. Therefore, an in-depth understanding of the molecular patterns of methionine metabolism in gliomas is crucial to science. As our group concluded in a previous study, the CBL-LSD1-CXCL8 signaling axis modulates intracellular lipid metabolism processes in methionine-restricted microenvironments and maintains cell survival [17]. In this study, further transcriptomic analysis of glioma cells tolerant to the methionine-restricted microenvironment revealed that the angiogenic signaling pathway was highly enriched and that CXCL8 was the most significantly up-regulated gene expressed by glioma cells in response to the methionine-restricted microenvironment. Based on the TCGA database, the CGGA database, and clinical tissue samples, CXCL8 expression was significantly higher in glioma tissues than in non-tumor tissues, and its expression was significantly higher in high-grade glioma tissues than in low-grade glioma tissues. Thus, CXCL8 is highly expressed in gliomas, and its level may be correlated with the degree of malignancy. High levels of CXCL8 have been found to induce epithelial-mesenchymal transition and promote the proliferation, migration and invasion of tumor cells through the JAK/STAT1/HIF-1α/Snail pathway [34]. CXCL8 plays a key role in angiogenesis, immune regulation, and other aspects of cerebral gliomas. High levels of CXCL8 can stimulate tumor angiogenesis or recruit immunosuppressive cells, while tumor-infiltrating neutrophils contribute to the progression of gliomas by regulating the HMGB1/RAGE/CXCL8 axis [35]. This study showed that high CXCL8 expression negatively correlated with survival in patients with glioma, suggesting that CXCL8 could be a potential biomarker of disease progression and prognosis.

In this study, we found that in the methionine-restricted microenvironment, the expression levels of CXCL8 and VEGFA in glioma cells were significantly elevated, as well as CD31 and VEGFA levels in grafted tumor tissues, and the length of tubule-forming tubules and the number of nodes of tubule generation were significantly increased. In contrast, after the inhibition of the expression of CXCL8 by SB225002, the angiogenesis of the CAM-grafted tumor in chicken embryos and the vascular density of the YSM in chicken embryos were significantly reduced, and the immunohistochemical results revealed significant reductions in CD31 and VEGFA expressions. Based on transcriptome sequencing analysis, CXCL8 regulates methionine metabolic reprogramming via energy metabolism regulatory mechanisms, including ATP synthesis-coupled electron transport, electron transport chain, oxidative phosphorylation, and nucleoside triphosphate metabolism. A blockade of the CXCL8–CXCR2 signaling axis inhibited glioma cell proliferation [17], and glioma angiogenesis. In accordance with our study, Burcu also found that VEGFA and CXCL8 were significantly overexpressed in patients with glioma [36], and that both VEGFA and CXCL8 could promote angiogenesis and accelerate the progression of LGG to GBM. Consequently, CXCL8 may be an essential molecular therapeutic target for gliomas in the future. Further studies are needed to understand the interactions between genes and metabolites for the downstream of the CXCL8–CXCR2 signaling axis during the reprogramming of methionine metabolism.

In summary, this study explored the relationship between methionine metabolic reprogramming and angiogenesis through a methionine-restricted resistant glioma cell model and identified CXCL8 and the CXCL8–CXCR2 signaling axis as positive regulators of methionine metabolism to promote angiogenesis in gliomas. This study provides a new perspective for elucidating the molecular mechanisms of resistance to antivascular therapy and highlights the potential that CXCL8 may have as a biomarker in antivascular treatment, providing new ideas for interventions of the CXCL8–CXCR2 signaling axis to improve antivascular therapy effectiveness.

## Supplementary Information

Additional file1 (PDF 7461 KB)

Additional file2 (XLSX 21 KB)

Additional file3 (XLSX 13 KB)

Additional file4 (XLSX 11 KB)

## Data Availability

The basic research data that support the findings of this study are available on request from the corresponding author, Lude Wang, upon reasonable request. The sequencing data that support the findings of this study are openly available in at https://xenabrowser.net/, http://www.cgga.org.cn/download.jsp, 10.3389/fcell.2021.633259 and 10.1016/j.cyto.2021.155789.
