# Supplementary material for: Mechanism of CXCL8 regulation of methionine metabolism to promote angiogenesis in gliomas
Source: Discov Oncol. 2024 Nov 2;15:614. doi: 10.1007/s12672-024-01467-2 (PMC11531453; doi:10.1007/s12672-024-01467-2)

Figure 3C. Original western blots for three repeats

Repeat1

MSM(day)

CXCL8 ~10KDa

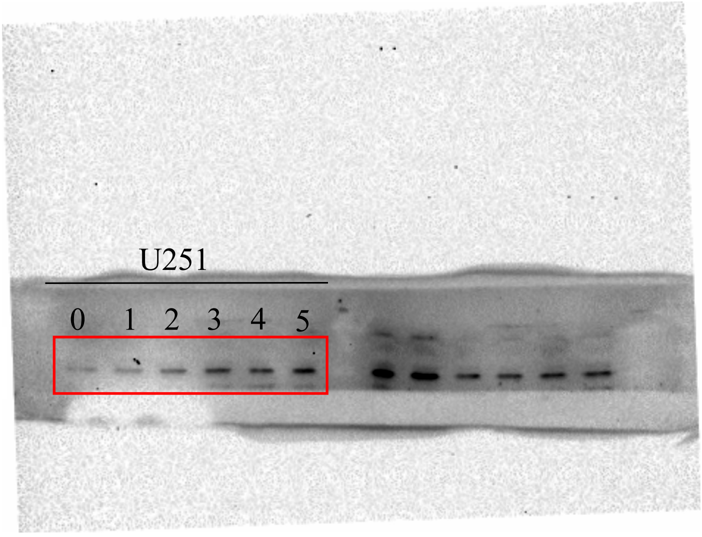


MSM(day)

β-actin ~40KDa

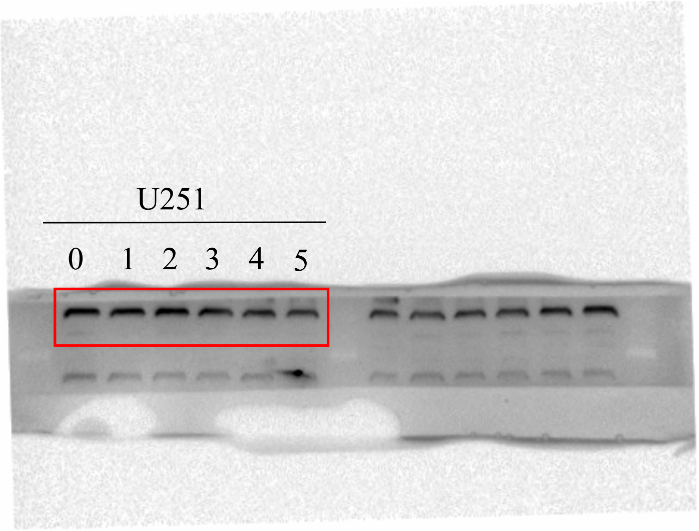

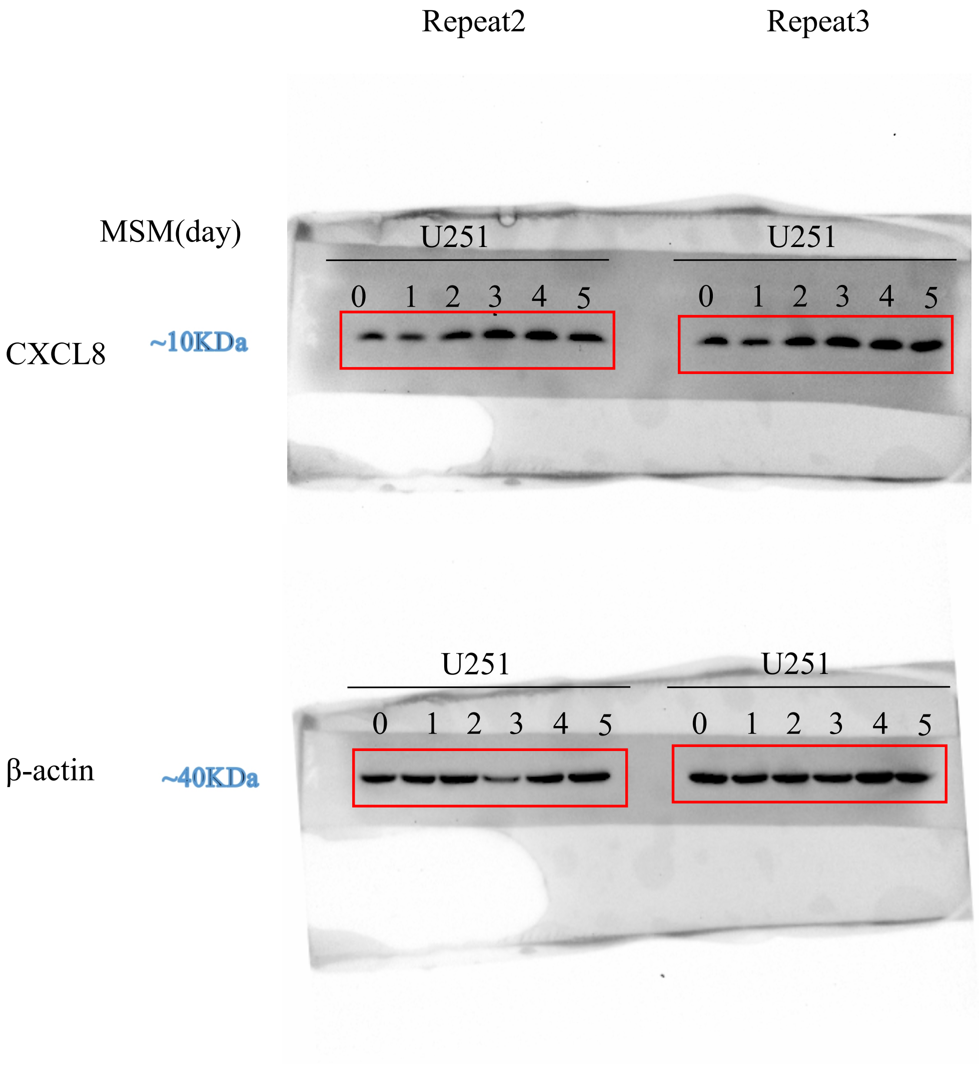
Repeat2
Repeat3
MSM(day)
U251
U251
0 1 2 3 4 5
0 1 2 3 4 5
CXCL8
~10KDa
U251
U251
0 1 2 3 4 5
0 1 2 3 4 5
β-actin
~40KDa

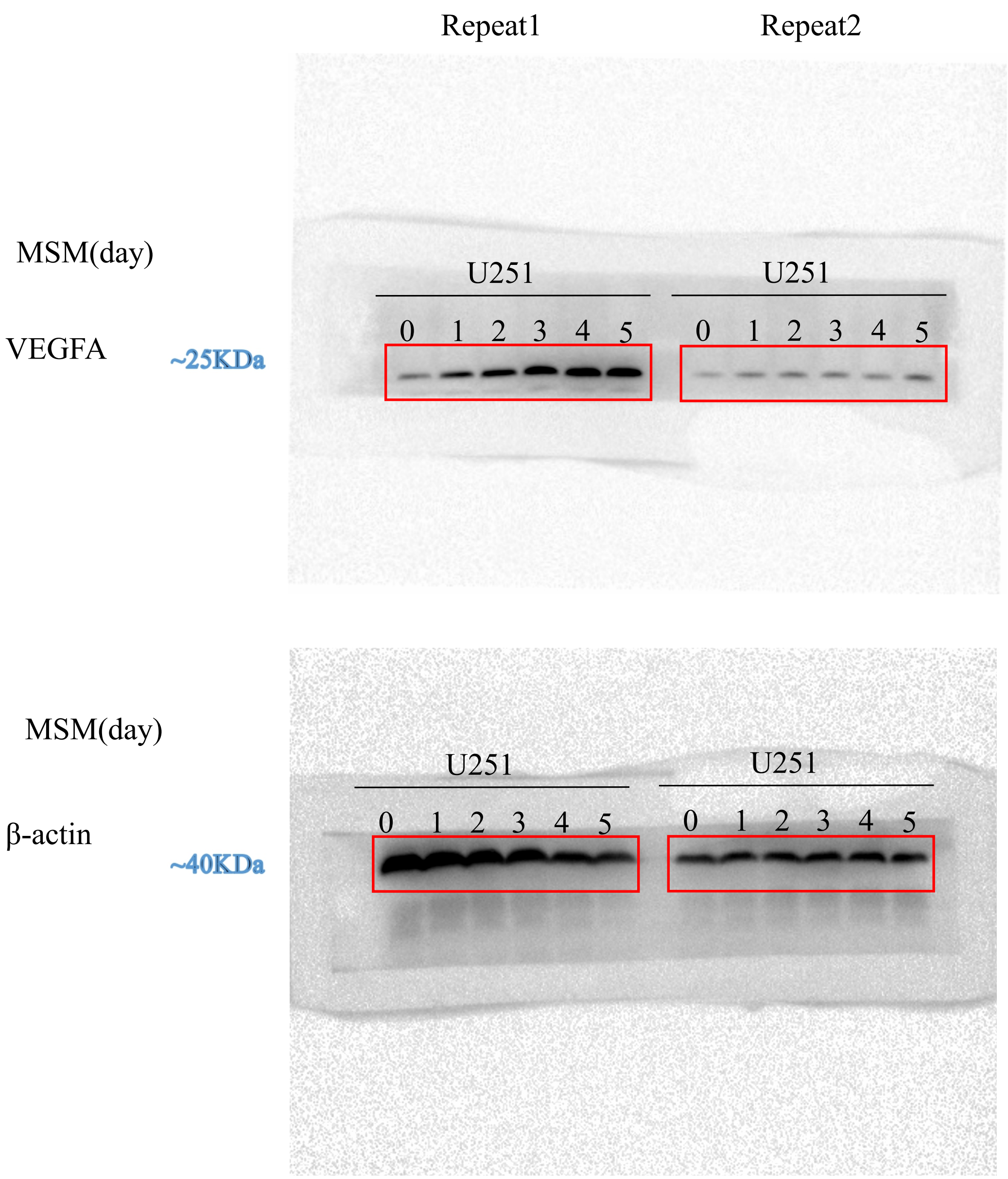
Repeat1
Repeat2
MSM(day)
U251
U251
0 1 2 3 4 5
0 1 2 3 4 5
VEGFA
~25KDa
MSM(day)
U251
U251
0 1 2 3 4 5
0 1 2 3 4 5
β-actin
~40KDa

Repeat3

MSM(day)

VEGFA ~25KDa

β-actin ~40KDa

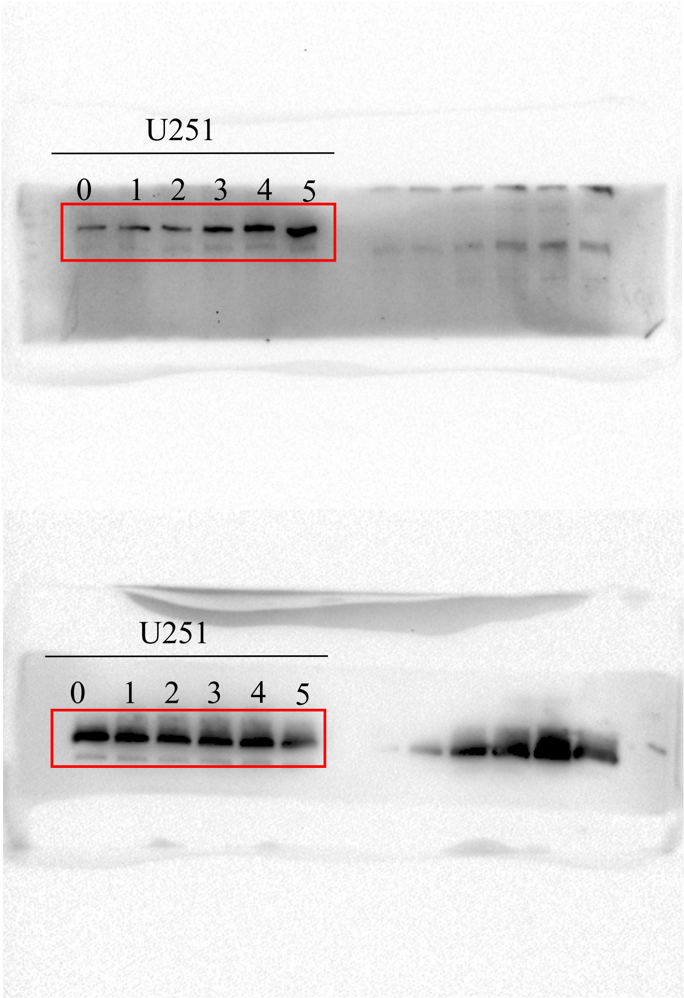

Supplement: Supplementary file 1 — Additional file1 (PDF 7461 KB) [file 12672_2024_1467_MOESM1_ESM.pdf]
